# Effects of Neuroendocrine CB1 Activity on Adult Leydig Cells

**DOI:** 10.3389/fendo.2016.00047

**Published:** 2016-06-03

**Authors:** Gilda Cobellis, Rosaria Meccariello, Rosanna Chianese, Teresa Chioccarelli, Silvia Fasano, Riccardo Pierantoni

**Affiliations:** ^1^Dipartimento di Medicina Sperimentale, Seconda Università di Napoli, Napoli, Italy; ^2^Dipartimento di Scienze Motorie e del Benessere, Università di Napoli Parthenope, Napoli, Italy

**Keywords:** CB1, endocannabinoids, Leydig cells, steroidogenesis, spermatogenesis, testis

## Abstract

Endocannabinoids control male reproduction acting at central and local level *via* cannabinoid receptors. The cannabinoid receptor CB1 has been characterized in the testis, in somatic and germ cells of mammalian and non-mammalian animal models, and its activity related to Leydig cell differentiation, steroidogenesis, spermiogenesis, sperm quality, and maturation. In this short review, we provide a summary of the insights concerning neuroendocrine CB1 activity in male reproduction focusing on adult Leydig cell ontogenesis and steroid biosynthesis.

## Introduction

Since the identification and the cloning of cannabinoid receptor 1 (CB1) from mammalian brain (1, 2), the deep involvement of CB1 signaling in several physiological functions emerged (3, 4). Besides central control of processes, such as reproduction, neuroendocrine functions, stress, food intake, neurogenesis, memory, thermogenesis, and pain (3–7), CB1 exerts direct activity in male and female reproductive and non-reproductive cells and tissues (3, 4, 8–11). This membrane G-protein-coupled receptor has the ability to bind endogenous cannabinoids [i.e., anandamide (AEA) and 2-arachidonoylglycerol (2-AG)], phytocannabinoids [i.e., Δ^9^-tetrahydrocannabinol (THC), the main psychoactive constituent of *Cannabis sativa*] and synthetic antagonists and agonists [i.e., SR141716 and HU-210, respectively] (8, 12). Its activity strongly depends on ligand availability whose tone is endogenously modulated by the fatty acid amide hydrolase (FAAH). Here, we provide a brief summary of the insights concerning direct/indirect effects of CB1 activity on adult Leydig cell (ALC) ontogenesis and steroid biosynthesis.

## CB1 Activity and Ontogenesis of ALC

CB1 is expressed and functionally active early during the development, when it regulates embryonic and trophoblast stem cell survival, and the differentiation of several adult specialized tissues (13). CB1 activity has been shown to regulate proliferation and differentiation of mesoderm-derived mesenchymal stem cells, with a key role in cellular differentiation of several peripheral tissues including adipocytes, osteoblasts, skeletal muscle, and epithelial cells (13). A key role for CB1 has further emerged in ALC development, in both rat and mouse (14). Ontogenesis of these cells occurs postnatally from undifferentiated mesenchymal-like stem cells localized in the interstitial compartment of testis. Four distinct stages of the ALC development have been identified and characterized: (i) the stem Leydig cells, i.e., spindle-shaped interstitial mesenchymal cells able of self-renewal, differentiation, and replenishment of the Leydig cell niche; (ii) the progenitor Leydig cells deriving from stem Leydig cells and differentiating into (iii) immature Leydig cells; and (iv) the mature/ALCs originating from the immature ones through a single proliferation cycle (15). During postnatal development of rat testis (14), CB1 is expressed in the interstitial compartment from the early postnatal days. Later, with the appearance of round spermatids, CB1 is localized in the tubular compartment. In interstitial cells, CB1 expression is related to stem and/or progenitor cells committed to differentiate into immature Leydig cells. Its expression shuts down in immature Leydig cells when these cells undergo proliferation to produce fully developed ALC (14). Afterward, CB1 results to be stably expressed in these steroid-secreting cells suggesting a direct effect in the modulation of steroidogenesis. In agreement with these observations, CB1 activation inhibits basal testosterone secretion *in vivo* and *in vitro* (16), while its inactivation promotes the downregulation of neuroendocrine axis, decrease of testosterone and estradiol levels, and the development of few ALC as well, thus suggesting an involvement of CB1 in steroidogenesis and ALC ontogenesis (14, 16–19). Interestingly, as observed in adipocytes during their differentiation (13), CB1 seems to be positively related to differentiation events supervising the ontogenesis of Leydig cells and negatively with respect to their proliferation.

## CB1 Activity and Production of Sex Hormones

Hormonal milieu and a set of local modulators sustain the progression of spermatogenesis and the formation/release of high quality sperm (20). Thus, the testicular biosynthesis of testosterone – the classical male hormone – primarily requires the release of hypothalamic gonadotropin-releasing hormone (GnRH), which in turn induces the release in the bloodstream of pituitary gonadotropins (21).

CB1 has a leading role in the production of sex hormones; both THC and AEA repress testosterone secretion *via* inhibition of GnRH and gonadotropins synthesis/release (3, 10, 11, 16, 22–29), whereas sex steroids affect CB1 expression and endocannabinoid levels in the anterior pituitary gland (30). As a consequence, inhibitory effects on neuroendocrine axis (10, 11, 18), sexual behavior (28), and sperm quality (3, 9, 18, 19, 31–34) have been observed. In testis, besides spermatids and Sertoli cells (14, 34, 35), CB1 is expressed in the steroid-secreting ALC (14, 16, 36), thus suggesting direct effects on the modulation of ALC activity. Consistently, CB1 and the endovanilloid receptor TRPV1 exert direct, but opposite, effects on testicular GnRH signaling (36, 37), one of the main testicular bioregulators of spermatogenesis and steroidogenesis (21, 38, 39).

Thus, efforts to define the detailed molecular mechanisms to clarify how endocannabinoids regulate testosterone production in vertebrates have been made. In this respect, data from the anuran amphibian *Pelophylax esculentus* recently added insights in this intriguing story. In fact, *in vitro* incubations of frog testis and *in vivo* treatment with AEA have been carried out to analyze the expression of cytochrome P450 17α-hydroxylase/17,20 lyase (*cyp17*) and 3β-hydroxysteroid dehydrogenase/D-5-4 isomerase (3β*-HSD*), key enzymes in sex steroid biosynthesis. Interestingly, the *in vivo* treatment only had an effect on steroidogenesis, thus suggesting that the functionality of the hypothalamus–pituitary axis is essential to support the role of endocannabinoids in the regulation of steroidogenesis in amphibians (40). However, the decrease of *cyp17* may involve receptors other than CB1 since SR141716 administration does not restore its expression to control level.

Since CB1 has the ability to regulate, directly or indirectly, the activity of hypothalamic GnRH [see review in Ref. (29, 11)] in different cell lines (41), as well as in mammalian (24, 42) and non-mammalian vertebrate species (25, 26, 43), and endocannabinoids are in sharp contrast with kisspeptins, the emerging positive regulators of GnRH system at both central and testicular level (44, 45). Their involvement in the control of ALC activity (45–47), in the regulation of estrogen-dependent reproductive functions (47), and spermatogenesis progression (47) has recently been suggested. Hence, the last insight in CB1 activity and testosterone production is considering kisspeptin system as new cannabinoid target in the hypothalamus. In fact, CB1 activation induces the downregulation of *kiss1*, the gene encoding kisspeptin, in male rats under stress condition (48) but also in frog testis, under physiological conditions (49). In particular, in frog, the molecular mechanism has recently been elucidated. In fact, *in vivo* administration of AEA, *via* CB1, decreases the transcription level of GnRH, ligands and receptors, in the diencephalon, but also negatively affects Kiss1 and kisspeptin receptor GPR54 in both diencephalon and testis. As a consequence, intratesticular testosterone level decreases and estradiol production increases due to the parallel increase of the P450 aromatase (*cyp19*) mRNA/protein (49). Therefore, the endocrine route well integrates with the local circuitry that modulates AEA tone by FAAH, the major checkpoint of endocannabinoid signaling in vertebrates occurring *via* estrogen biosynthesis in both mammalian and non-mammalian testis (49–51). In fact, *in vitro* incubations with AEA or 17β-estradiol both increase the FAAH levels, and AEA effects are fully counteracted by the anti-estrogen ICI182780 (49). Therefore, high AEA tone, *via cyp19*, induces estradiol biosynthesis with subsequent FAAH production and AEA hydrolysis. Thus, AEA may control testis physiology – centrally – acting through kisspeptin system and – locally – modulating its tone *via* the biosynthesis of estradiol, which in turn targets FAAH protein (49). The direct activity of AEA exerted *via* CB1 on *cyp19* is confirmed by the characterization of *CB1*^−/−^ mice, a model that efficiently synthesizes LH but shows low LH, testosterone, and estradiol levels in the bloodstream (16, 18, 19). Besides the impairment of GnRH signaling (18, 19) and the fewer ALC (14) responsible for LH drop and testosterone depletion, respectively, *CB1*^−/−^ mice express low *cyp19*, but normal *3*β*-HSD* mRNA levels (18, 19), suggesting a direct CB1 activity in the control of estradiol biosynthesis.

## Closing Remarks

The use of mammalian and non-mammalian animal models should be considered useful tool for studying the involvement of endocannabinoids in the regulation of evolutionarily conserved biological processes such as spermatogenesis (25, 39, 52). Endocannabinoid system has been characterized in Sertoli and germ cells, while very few information about ALC are available. Since the central role played by ALC in the regulation of testicular activity *via* endocannabinoid system, we argue that studies should be addressed in this direction.

## Author Contributions

GC and RM: conception and design of the work, critical revision, and final version approval; RC and TC: manuscript drafting; and SF and RP: final version approval.

## Conflict of Interest Statement

The authors declare that the research was conducted in the absence of any commercial or financial relationships that could be construed as a potential conflict of interest.
